# Head Color Morph‐ and Sex‐Specific Differences in Follistatin Gene Expression in the Gouldian Finch Brain

**DOI:** 10.1002/cne.70098

**Published:** 2025-10-17

**Authors:** Changjiu Zhao, Farrah N. Madison

**Affiliations:** ^1^ Department of Integrative Biology University of Wisconsin–Madison Madison Wisconsin USA

**Keywords:** activin, GraphPad Prism (RRID:SCR_002798), polymorphism, QuPath software (RRID:SCR_018257), sex differences, songbird, Zebrafinch Brain Architecture Project (RRID:SCR_004277)

## Abstract

The Gouldian finch exhibits a head color polymorphism, which is tightly coupled to distinct differences in aggression, stress responses, and parental care. In competitive environments, red‐headed birds are more aggressive, are less parental, and exhibit a heightened stress response relative to black‐headed birds. The head color polymorphism has been associated with genetic variation in a small noncoding region near the follistatin (*FST*) gene. Given the regulatory nature of this gene, we hypothesized that *FST* mRNA would be differentially expressed in association with morph‐ and sex‐specific differences in the brains of red‐ and black‐headed morphs. To test this hypothesis, we analyzed *FST* gene expression in the Gouldian finch brain using RNAscope in situ hybridization assay. Our results revealed significant differences in *FST* gene expression between morphs and sex. Specifically, black‐headed morphs, regardless of sex, displayed higher *FST* mRNA levels across multiple brain regions associated with aggression, stress responses, and parental care compared to red‐headed morphs. Furthermore, males consistently showed greater *FST* mRNA levels within the same morph type than females. These findings suggest that head color morph‐ and sex‐specific differences in *FST* gene expression may underlie the observed morph‐ and sex‐specific differences in aggression, stress responses, and parental care in Gouldian finches.

## Graphical abstract

Using highly sensitive and specific RNAscope in situ hybridization, we revealed that black‐headed morphs displayed higher follistatin (*FST*) mRNA levels across multiple brain regions associated with aggression, stress responses, and parental care compared to red‐headed morphs. Males consistently showed greater *FST* mRNA levels within the same morph type than females. These findings suggest that head color morph‐ and sex‐specific differences in *FST* gene expression may underlie the observed morph‐ and sex‐specific differences in aggression, stress responses, and parental care in Gouldian finches.

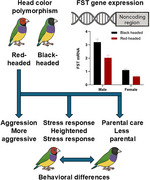

## 1 Introduction

Genetic‐based color polymorphisms, where multiple discrete color morphs occur within a population, are often associated with morph‐specific behavioral and physiological strategies (Hugall and Stuart‐Fox 2012). The genetic basis of these color variations frequently lies in a small number of genes with large effects, which can lead to distinct phenotypic traits associated with specific adaptive strategies (McKinnon and Pierotti 2010; Roulin 2004). Such polymorphisms enable individuals within a population to exploit diverse ecological niches and social environments, thus maintaining genetic diversity.

Research in animal models such as lizards (Andrade et al. 2019), songbirds (Horton et al. 2012; Kim et al. 2019; Toomey et al. 2018), and fish (Maan and Sefc 2013) has established genetic‐based color polymorphisms as valuable tools for elucidating the genetic basis of complex social behaviors. For example, the common wall lizard exhibits a genetically linked polymorphism with three color morphs, white, orange, or yellow, linked to two small regulatory genes that have pleiotropic effects on behavior and hormones (Andrade et al. 2019). Similarly, in the white‐throated sparrow, a genetic‐based color polymorphism is linked to behavioral phenotypes, providing a direct connection between a set of specific genes and behaviors (Thomas et al. 2008). White morphs tend to display greater territorial aggression, risk‐taking behavior, and mate‐guarding, whereas tan morphs are less reactive and engage more in parental care (Thomas et al. 2008). In African cichlid fish, morph‐specific behavioral differences have also been observed, with yellow male morphs expressing higher levels of aggression compared to blue morphs (Maan and Sefc 2013). As alluded to above, these examples highlight that genetic polymorphisms associated with color polymorphisms can influence social behaviors across diverse taxa.

The Gouldian finch (*Chloebia gouldiae*) exhibits stable and distinct behavioral phenotypes tightly coupled to marked differences in aggression, parental care, immune function, and risk‐taking behaviors (Pryke and Griffith 2009; Pryke et al. 2007; Pryke and Griffith 2006). Red‐headed birds are more aggressive, less parental, and more neophobic than black‐headed birds (Pryke and Griffith 2006; Pryke 2007). Red‐headed morphs also exhibit low immune responsiveness in response to social competition, whereas black‐headed morphs remain largely unaffected (Pryke and Griffith 2009; Pryke et al. 2007). In addition to behavioral phenotypes, endocrine responses differ significantly between red‐ and black‐headed morphs. In response to socially competitive environments, circulating testosterone (a steroid hormone positively correlated with aggression) concentrations significantly increased in red‐headed birds while declining significantly in black‐headed birds (Pryke et al. 2007). Red‐headed morphs also exhibited a dramatic increase in circulating corticosterone (a steroid hormone positively associated with stress), while black‐headed morphs demonstrated no increase in corticosterone concentrations (Pryke et al. 2007, 2012). In another study, in which bound and free (i.e., biologically active) corticosterone were measured, when exposed to nutritional stress, red‐headed males exhibited a reduced level of free corticosterone and elevated corticosterone‐binding globulin (CBG) concentrations, whereas black‐headed morphs showed reduced CBG concentrations and increased levels of free corticosterone (Pryke et al. 2012). These distinct endocrine responses in the two morphs may underlie morph‐specific differences in social behavior.

Recent sequencing studies on the genetic basis of head feather color in red‐ and black‐headed Gouldian finch morphs identified a candidate locus within a small (∼70 kb) noncoding region mapping to the Z chromosome near the Follistatin (*FST*) gene (Kim et al. 2019; Toomey et al. 2018). FST is a multifunctional regulatory protein encoded by the *FST* gene. FST, as an activin‐binding protein, exerts its vast majority of biological functions in the CNS through antagonizing the action of the protein activin (Patel 1998; Phillips and de Kretser 1998). It is well‐known for regulating steroid hormones through suppressing follicle‐stimulating hormone (FSH) in the pituitary gland (Patel 1998). FST is also known for its involvement in a wide range of biological processes, such as muscle growth, energy metabolism, and reproduction (Patel 1998; Phillips and de Kretser 1998; Tsuchida 2006; Hansen and Plomgaard 2016).

In addition to the periphery, FST is widely expressed in the brain, and neural FST is receiving attention for its role in the hippocampus in neurogenesis, spatial learning, working memory, and long‐term potentiation (LTP) (Ageta et al. 2008; Chen et al. 2021). In mammals, FST is also expressed in brain regions associated with aggression, stress responses, and parental care (Macconell et al. 1996; Ogawa et al. 2020). These findings suggest a potential role for *FST* in social behavior and raise the possibility that differential expression of *FST* in the brains of red‐ and black‐headed morphs might be a genetic mechanism through which *FST* regulates morph‐specific differences in multiple behavioral and physiological processes.

To provide insight into this hypothesis, in this study, we used RNAscope in situ hybridization (ISH) to determine the extent to which *FST* mRNA is differentially expressed in red‐ and black‐headed morphs in 10 brain regions involved in aggression, stress responses, and parental care, where FST is also expressed in mammals.

## 2 Materials and Methods

### 2.1 Animals and Housing

Sixteen age‐matched Gouldian finches native to Australia (*n* = 8 males: 4 black‐headed, 4 red‐headed; *n* = 8 females: 4 black‐headed, 4 red‐headed) were purchased from an avian breeder and housed and maintained on a photoperiod of 12L:12D (light:dark). Birds were housed in an indoor aviary in 49 × 95 × 51‐cm same‐sex and mixed‐morph cages (six birds per cage) at the University of Wisconsin–Madison. Food and water were provided ad libitum, decreasing competition for resources. Birds were housed in these conditions for at least 6 months prior to tissue collection. All procedures and protocols were in accordance with a protocol approved by the University of Wisconsin–Madison Institutional Animal Care and Use Committee (IACUC).

### 2.2 Brain Tissue Processing

Brains were extracted, and tissue was flash‐frozen on crushed dry ice and stored at −80°C. Coronal sections at 16 µm thickness were directly mounted on Superfrost Plus Slides (Fisher Scientific, Cat. #22037246) using a cryostat (Leica CM1950) and stored at −80°C until labeling. To prepare sections for RNAscope ISH, sections were fixed in pre‐chilled fresh 10% buffered formalin (Fisher Scientific, Cat. #SF100‐4) for 60 min at 4°C. Sections then underwent serial dehydration in 50% (5 min), 70% (5 min), and 100% ethanol (2 × 5 min).

### 2.3 RNAscope ISH Assay

We used RNAscope fluorescent multiplex assay (Advanced Cell Diagnostics [ACD], Newark, CA) to investigate *FST* mRNA expression. We followed the protocol for RNAscope multiplex fluorescent v2 assay by ACD (Document #UM323100). Briefly, after drying slides at room temperature (RT) for 10 min, a hydrophobic pen was used to create a hydrophobic barrier around each section and allowed to dry at RT for 10 min. RNAscope hydrogen peroxide was applied to the sections and incubated at RT for 10 min. Following two washes with dH_2_O, sections were treated with RNAscope Protease IV and incubated at RT for 30 min. Sections were hybridized with Probe‐*FST*‐C1 (follistatin, channel 1) in the HybES hybridization oven and incubated at 40°C for 2 h. The probes designed and produced by ACD contain 20 signal‐generating oligo‐pairs targeting 296–1285 sequences of the *FST* gene. After hybridization, sections were subjected to a series of amplification buffers in the order of AMP1 at 40°C for 30 min, AMP2 at 40°C for 30 min, and AMP3 at 40°C for 15 min. Following signal amplification, sections were incubated in RNAscope Multiplex FL v2 HRP‐C1 at 40°C for 15 min. Tyramide signal amplification (TSA) vivid fluorophore 570 was added on each section for labeling the C1 probe (*FST*) and incubated at 40°C for 30 min. Sections were treated with RNAscope Multiplex FL v2 HRP Blocker at 40°C for 15 min. Sections were counterstained with DAPI, then coverslipped using ProLong Gold anti‐fade mountant (Invitrogen, P36930). Slides were allowed to dry in a dark hood overnight before being stored at 4°C. Images were captured within 1 week. RNAscope positive (targeting the cyclophilin B [PPIB] gene in zebra finch) and negative (targeting the dihydrodipicolinate reductase [DapB] gene from the *Bacillus subtilis* strain SMY, a soil bacterium) control probes were included. High positive signals were observed with PPIB probes, and no background staining was detected with DapB probes.

### 2.4 Quantification of *FST* mRNA Expression

**FIGURE 1:**
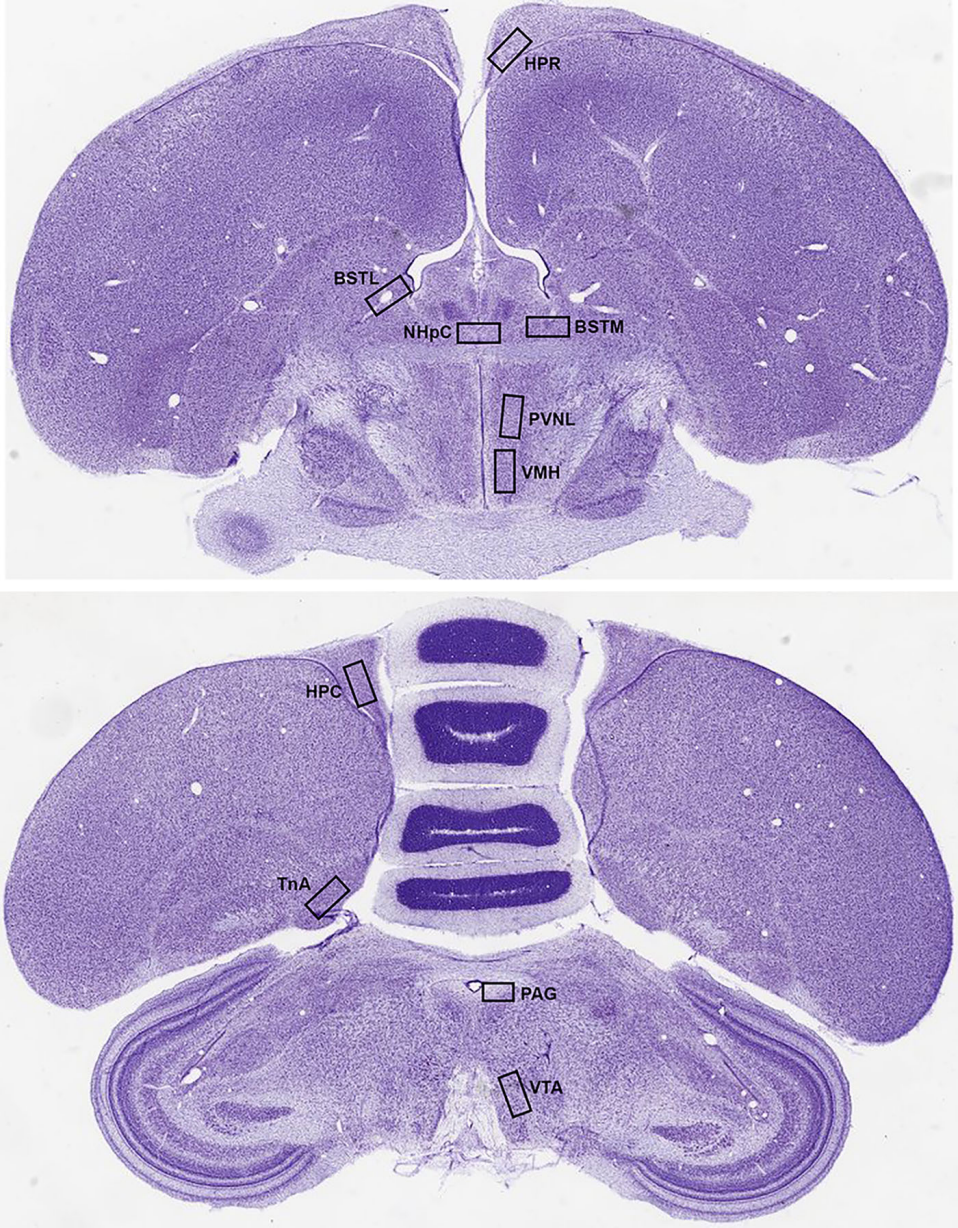
Coronal photomicrographs of a Nissl‐stained Gouldian finch where the brain regions (boxed areas) were examined and quantified for follistatin (*FST*) mRNA expression. Images were adapted and modified from Zebrafinch Brain Architecture Project (RRID:SCR_004277; URL: http://zebrafinch.brainarchitecture.org). BSTL, lateral bed nucleus of the stria terminalis; BSTM, medial bed nucleus of stria terminalis; FST, follistatin; HPC, caudal hippocampal formation, hippocampus; HPR, rostral hippocampal formation, hippocampus; NHpC, nucleus of the hippocampal commissure; PAG, periaqueductal gray; PVNL, lateral part of the paraventricular nucleus; TnA, nucleus taeniae of the amygdala; VMH, ventromedial nucleus of the hypothalamus; VTA, ventral tegmental area.

The RNAscope ISH assay generates punctate dots each representing a single mRNA molecule. We quantified *FST* mRNA expression by calculating the average number of punctate dots per cell. All images were acquired with a screen resolution of 1024 × 1024 pixels and 40x magnification using an inverted Zeiss LSM 710 Meta laser scanning confocal microscope (Zeiss; Oberkochen, Germany). Counting of punctate dots was carried out in 10 brain regions known to be involved in stress responses, aggression, and parental care (Goodson et al. 2005, 2012; Fokidis et al. 2013; Nagarajan et al. 2017, 2014; Goodson and Evans 2004), including the lateral part of the paraventricular nucleus (PVNL) (205 × 315 µm) and ventromedial nucleus of the hypothalamus (VMH) (205 × 315 µm), medial bed nucleus of the stria terminalis (BSTM) (200 × 200 µm), lateral bed nucleus of the stria terminalis (BSTL) (180 × 180 µm), rostral hippocampal formation (HPR) (150 × 250 µm) and caudal hippocampal formation (HPC) (150 × 250 µm), nucleus of the hippocampal commissure (NHpC) (200 × 150 µm), periaqueductal gray (PAG) (250 × 200 µm), nucleus taeniae of the amygdala (TnA) (250 × 200 µm), and ventral tegmental area (VTA) (205 × 270 µm). We additionally planned to quantify *FST* mRNA in the lateral septum (LS) and medial preoptic area (MPOA) given the established roles for these regions in aggression and parental behavior (Lischinsky and Lin 2020; Numan and Insel 2003; Kohl and Dulac 2018); however, little to no label was observed in these regions. In each bird, the number of punctate dots including clusters (e.g., overlapping dots from multiple mRNA molecules) was counted unilaterally in a typical section that was anatomically well‐matched across all animals (Figure 1). Simultaneously, DAPI‐positive nuclei were also enumerated in the same counting area. The counting was performed by an experienced investigator unaware of the animal conditions using QuPath software v0.5.1 (Open software for Bioimage analysis, RRID:SCR_018257, URL: https://qupath.github.io). The average number of punctate dots per cell from each target region was obtained by dividing the number of punctate dots by the number of DAPI‐positive nuclei.

### 2.5 Statistical Analysis

Statistical analyses were performed using GraphPad Prism Software (RRID:SCR_002798; version 10.1.0; GraphPad Software, San Diego, CA). The number of average punctate dots per cell was presented as mean ± *SEM* and analyzed using two‐way analysis of variance (ANOVA) with sex (two levels: male and female) × morph (two levels: black‐headed and red‐headed) as between‐subject factors. When overall significant effects were found, pairwise comparisons of means were assessed using Fisher's LSD post hoc test. The number of cells per target region (cell density) was presented as mean ± *SEM* and analyzed using one‐way ANOVA. When overall significant effects were found, pairwise comparisons of means were assessed using Bonferroni post hoc test. Differences were considered significant at *p* ≤ 0.05.

## 3 Results

### 3.1 General Expression Patterns of *FST* mRNA in the Gouldian Finch Brain

**FIGURE 2:**
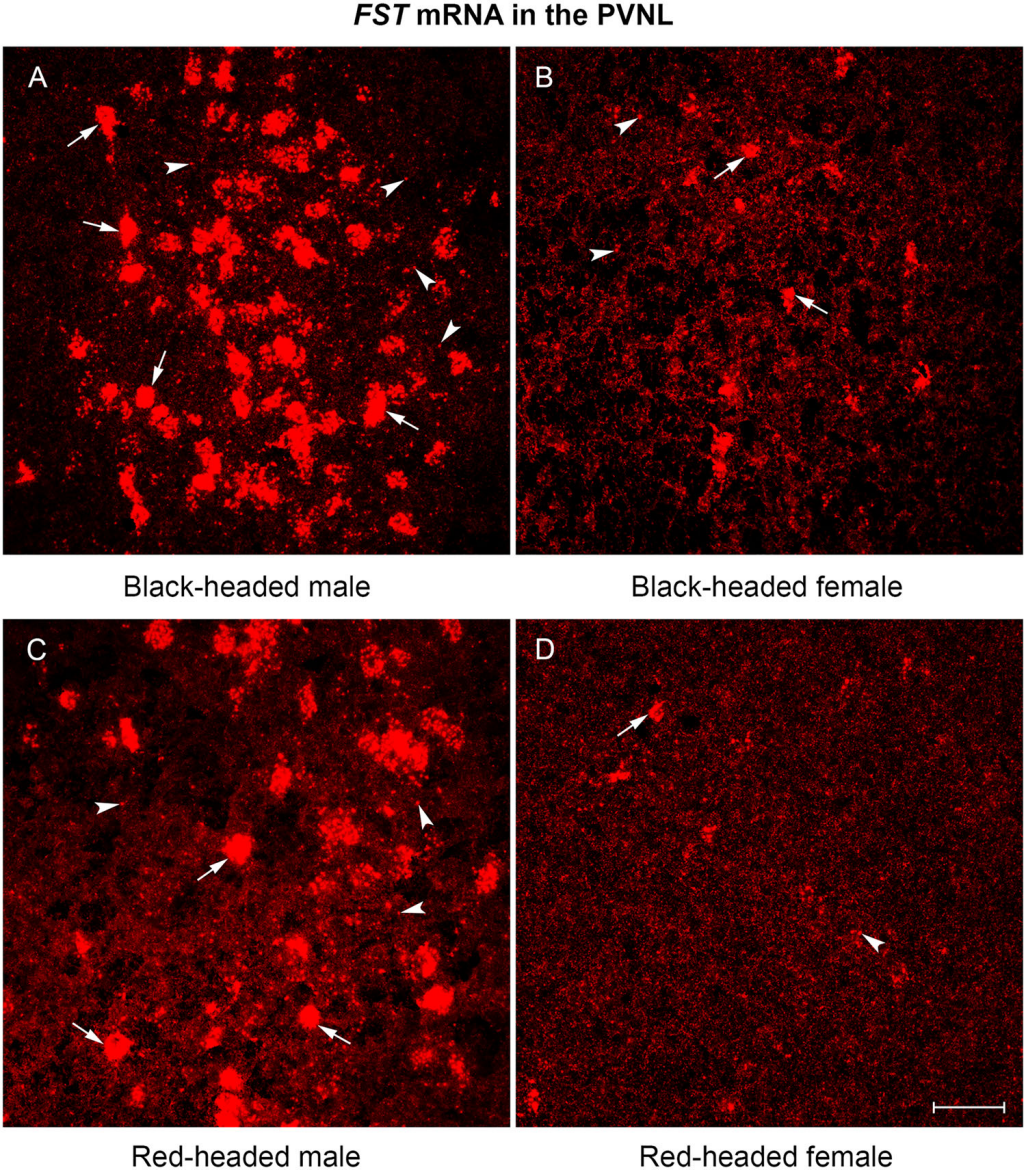
Representative photomicrographs of *FST* mRNA expression in the PVNL of Gouldian finch brain. (A) Black‐headed male. (B) Black‐headed female. (C) Red‐headed male. (D) Red‐headed female. Note that many *FST* mRNA molecules formed large clusters in both black‐ and red‐headed males. Arrows indicate clusters, and arrowheads indicate punctate dots, each representing a single mRNA molecule. FST, follistatin; PVNL, lateral part of the paraventricular nucleus. Scale bar: 50 µm.

**FIGURE 3:**
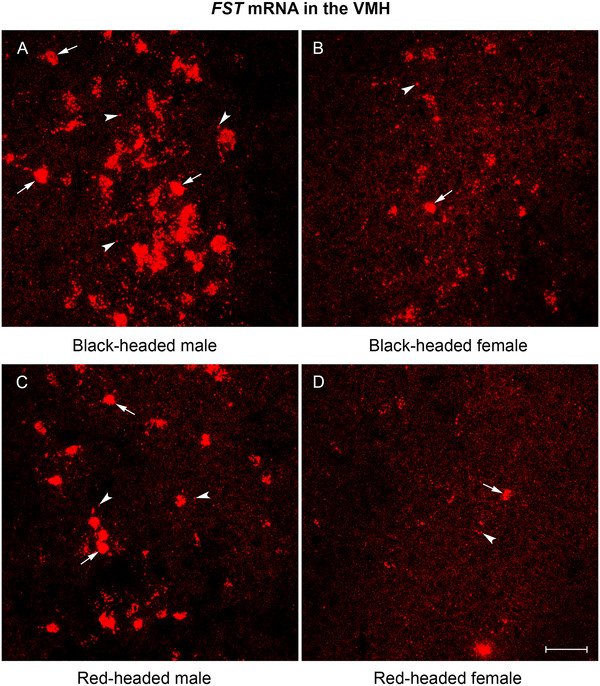
Representative photomicrographs of *FST* mRNA expression in the VMH of Gouldian finch brain. (A) Black‐headed male. (B) Black‐headed female. (C) Red‐headed male. (D) Red‐headed female. Note that in both black‐ and red‐headed males, numerous *FST* mRNA molecules formed large clusters. Arrows indicate clusters, and arrowheads indicate punctate dots. FST, follistatin; VMH, ventromedial nucleus of the hypothalamus. Scale bar: 50 µm.

**FIGURE 4:**
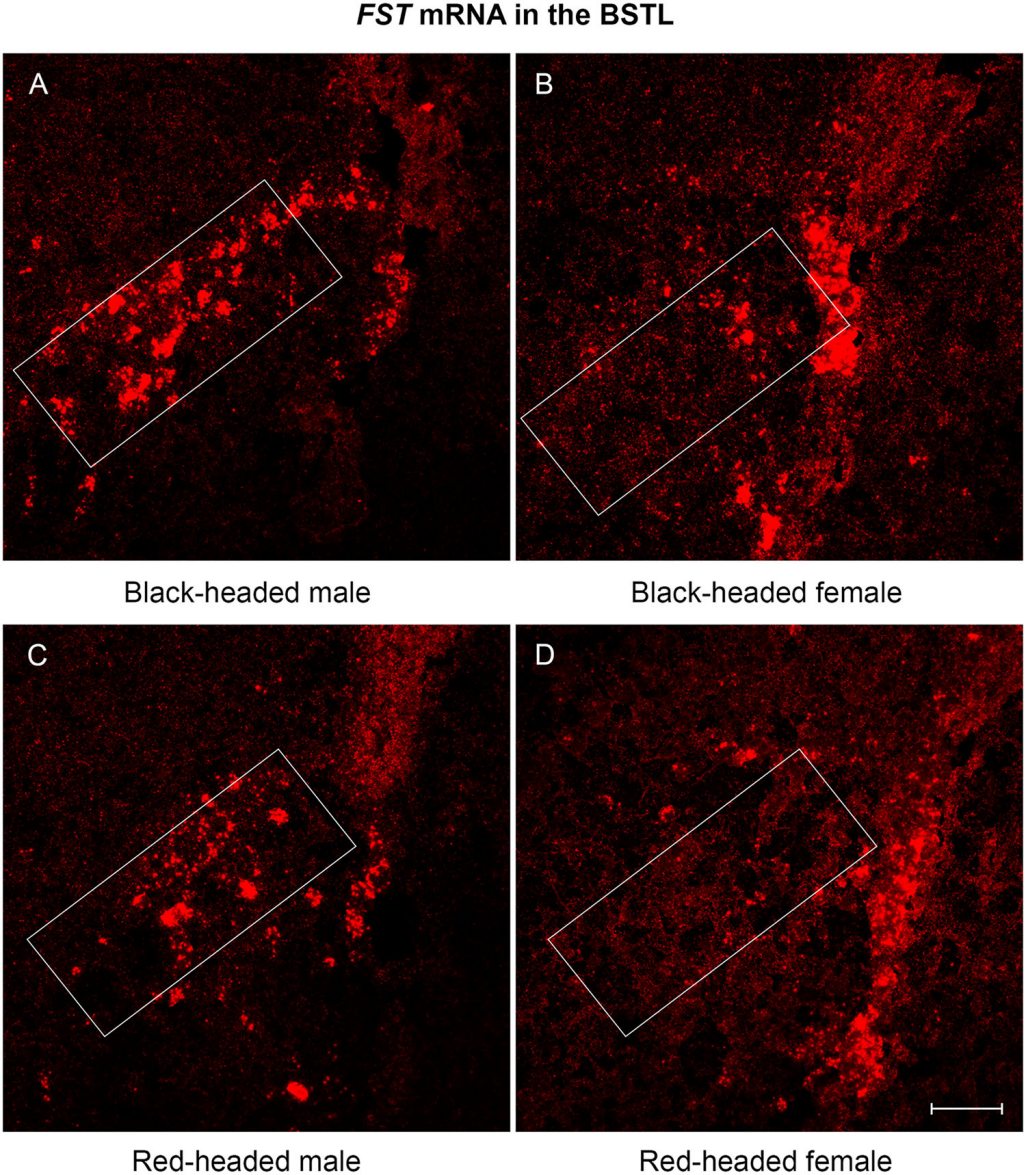
Representative photomicrographs of *FST* mRNA expression in the BSTL of Gouldian finch brain. (A) Black‐headed male. (B) Black‐headed female. (C) Red‐headed male. (D) Red‐headed female. *FST* mRNAs were quantified in the white boxed areas. FST, follistatin; BSTL, lateral bed nucleus of the stria terminalis. Scale bar: 50 µm.

Among the 10 brain regions quantified, *FST* mRNA expression was unevenly distributed. *FST* mRNA was abundantly expressed in the PVNL, VMH, and BSTL of black‐ and red‐headed males (Figures 2, 3, and 4A,C). In the hippocampus, higher levels of *FST* mRNA expression were observed in the HPR compared to the HPC (Figure 6E,J). Moderate levels of *FST* mRNA expression were present in the NHpC, PAG, and VTA. As mentioned in the methods, *FST* mRNA was absent from the LS and MPOA and thus not quantified.

### 3.2 Overall *FST* mRNA Expression in the Gouldian Finch Brain

Of the 10 brain regions examined, seven (PVNL, VMH, BSTM, BSTL, PAG, VTA, and HPC) revealed a significant effect of sex, six showed a significant effect of morph (PVNL, VMH, BSTM, BSTL, PAG, and VTA), and three indicated a significant interaction between sex and morph (PVNL, VMH, and BSTM). No significant ANOVA results were obtained for HPR, NHpC, and TnA.

### 3.3 Cell Density in Target Regions of the Gouldian Finch Brain

As shown in Table 1, there were no significant differences in the number of *FST*‐expressing cells in seven target regions between morphs and sex, including PVNL, BSTM, BSTL, HPR, PAG, VTA, and TnA. Noticeably, morph differences in the number of cells that express FST mRNAs were found in the VMH (black‐headed female vs. red‐headed female, *p* = 0.02), NHpC (black‐headed female vs. red‐headed female, *p* = 0.03), and HPC (black‐headed male vs. red‐headed male, *p* = 0.037). No sex differences were observed.

**TABLE 1:** Cell density in target brain regions.

Region	Black‐headed male (BM)	Black‐headed female (BF)	Red‐headed male (RM)	Red‐headed female (RF)
PVNL	252.0 ± 3.9	257.8 ± 6.6	264.0 ± 7.3	263.8 ± 5.5
VMH	267.8 ± 5.2	241.5 ± 7.5^*^	261.5 ± 5.9	273.0 ± 5.6
BSTM	134.5 ± 2.7	141.5 ± 2.1	136.3 ± 1.9	142.0 ± 2.9
BSTL	118.5 ± 3.0	119.5 ± 1.6	123.0 ± 2.8	123.3 ± 2.6
HPR	133.5 ± 5.1	124.5 ± 7.8	128.5 ± 5.6	131.5 ± 6.2
NHpC	155.5 ± 5.1	144.3 ± 4.3^*^	152.8 ± 5.2	185.0 ± 14.5
PAG	139.5 ± 7.3	147.0 ± 4.4	159.8 ± 6.4	151.8 ± 2.1
VTA	156.8 ± 13.4	178.5 ± 11.2	191.3 ± 8.5	200.5 ± 5.0
TnA	204.0 ± 6.8	201.3 ± 3.1	201.3 ± 3.3	201.8 ± 5.3
HPC	146.5 ± 3.2^*^	155.3 ± 3.5	170.0 ± 7.0	161.0 ± 5.4

*Note:* Data are presented as mean ± *SEM*. The number of cells in target brain regions was measured by counting DAPI‐positive nuclei.

*p = 0.02 (BF vs. RF for VMH); *p = 0.03 (BF vs. RF for NHpC); *p = 0.037 (BM vs. RM for HPC).

### 3.4 *FST* mRNA Expression in the PVNL

*FST* mRNA expression was mainly restricted to the lateral part of the PVN. *FST* mRNA molecules formed large, neuron‐like clusters (Figure 2A,C), which are overlapping signals from multiple mRNA molecules in close proximity to each other. A two‐way ANOVA revealed a significant effect for sex, *F*(1, 12) = 450.9, *p* < 0.0001; a significant effect for morph, *F*(1, 12) = 100.2, *p* < 0.0001; and a significant interaction between sex and morph, *F*(1, 12) = 18.04, *p* = 0.0011. Black‐headed males exhibited the highest level of *FST* mRNA expression, while red‐headed females displayed the lowest mRNA expression. In both males and females, *FST* mRNA expression in black‐headed birds was much higher when compared to red‐headed birds (Figures 2A–D and 6A; *p* < 0.0001 for males, *p* = 0.0015 for females). Furthermore, in both black‐headed and red‐headed morphs, males displayed greater *FST* mRNA expression compared to females (Figures 2A–D and 6A; both *p*‐values <0.0001).

### 3.5 *FST* mRNA Expression in the VMH

Similar to the expression pattern in the PVNL, *FST* mRNA in the VMH was differentially expressed among birds. Densely and intensely labeled punctate dots and large clusters were observed in black‐headed males (Figure 3A) as opposed to the sporadically and lightly labeled dots in red‐headed females (Figure 3D). A two‐way ANOVA revealed a significant effect for sex, *F*(1, 12) = 204.1, *p* < 0.0001; a significant effect for morph, *F*(1, 12) = 52.13, *p* < 0.0001; and a significant interaction between sex and morph, *F*(1, 12) = 8.52, *p* = 0.013. Regardless of sex, a higher level of *FST* mRNA was observed in black‐headed birds relative to red‐headed birds (Figures 3A–D and 6B; *p* < 0.0001 for male, *p* = 0.01 for female). In addition, regardless of head color morph, males displayed a higher level of *FST* mRNA in comparison to females (Figures 3A–D and 6B; both *p*‐values <0.0001).

### 3.6 *FST* mRNA Expression in the BSTM

A two‐way ANOVA revealed a significant effect for sex, *F*(1, 12) = 205.2, *p* < 0.0001; a significant effect for morph, *F*(1, 12) = 129.8, *p* < 0.0001; and a significant interaction between sex and morph, *F*(1, 12) = 19.98, *p* = 0.0008. Black‐headed male and female Gouldian finches exhibited a higher level of *FST* mRNA than that of red‐headed birds (Figure 6C; *p* < 0.0001 for male, *p* = 0.0004 for female). Similarly, in both black‐headed and red‐headed birds, a greater level of *FST* mRNA was observed in males than that of females (Figure 6C; both *p* values <0.0001).

### 3.7 *FST* mRNA Expression in the BSTL

A comparable level of *FST* mRNA to that in the PVNL was found in the BSTL. Dense and intense dot‐like signals with a few small clusters were present in the black‐headed males (Figure 4A). We noticed that the *FST* mRNA was not evenly distributed within the BSTL since more mRNA signals were observed in the lateral part of the BSTL (Figure 4A,C). A two‐way ANOVA revealed a significant effect for sex, *F*(1, 12) = 67.05, *p* < 0.0001; a significant effect for morph, *F*(1, 12) = 154.3, *p* < 0.0001; but no significant interaction between sex and morph. In both males and females, *FST* mRNA level was higher in black‐headed birds than red‐headed birds (Figures 4A–D and 6D; both *p*‐values <0.0001). Additionally, in both black‐headed and red‐headed birds, males displayed a higher level of *FST* mRNA than females (Figures 4A–D and 6D; *p* < 0.0001 for black‐headed, *p* = 0.0001 for red‐headed).

### 3.8 *FST* mRNA Expression in the HPR, NHpC, and TnA

*FST* mRNA was expressed at a weak to moderate level in the HPR, NHpC, and TnA. TnA, in which only sporadic mRNA signals were positively labeled, displayed the lowest expression among the 10 brain regions examined (Figure 6A–J). There was no significant difference in *FST* mRNA in the HPR (Figure 6E), NHpC (Figure 6F), and TnA (Figure 6I) when comparing sex and head color morph.

### 3.9 *FST* mRNA Expression in the PAG

Low levels of *FST* mRNA molecules were expressed in the PAG. Only punctate dot‐like signals were detected. A two‐way ANOVA revealed a significant effect for sex, *F*(1, 12) = 63.16, *p* < 0.0001; a significant effect for morph, *F*(1, 12) = 22.18, *p* = 0.0005; but no significant interaction between sex and morph. Regardless of sex, *FST* mRNA level was higher in black‐headed birds relative to red‐headed birds (Figure 6G; *p* = 0.0034 for male, *p* = 0.01 for female). Moreover, in both black‐headed and red‐headed birds, males displayed higher level of *FST* mRNA than females (Figure 6G; *p* < 0.0001 for black‐headed, *p* = 0.0002 for red‐headed).

### 3.10 *FST* mRNA Expression in the VTA

*FST* mRNA was expressed at a relatively low level in the VTA. A morphological feature in this region was the lack of clusters. A two‐way ANOVA revealed a significant effect for sex, *F*(1, 12) = 34.26, *p* < 0.0001; a significant effect for morph, *F*(1, 12) = 32.38, *p* = 0.0001; but no significant interaction between sex and morph. Black‐headed male and female Gouldian finches displayed a greater level of *FST* mRNA than red‐headed birds (Figure 6H; *p* = 0.027 for male, *p* = 0.0001 for female). Further, in both black‐headed and red‐headed birds, males displayed higher level of *FST* mRNA than females (Figure 6H; *p* = 0.021 for black‐headed, *p* = 0.0001 for red‐headed).

### 3.11 *FST* mRNA Expression in the HPC

**FIGURE 5:**
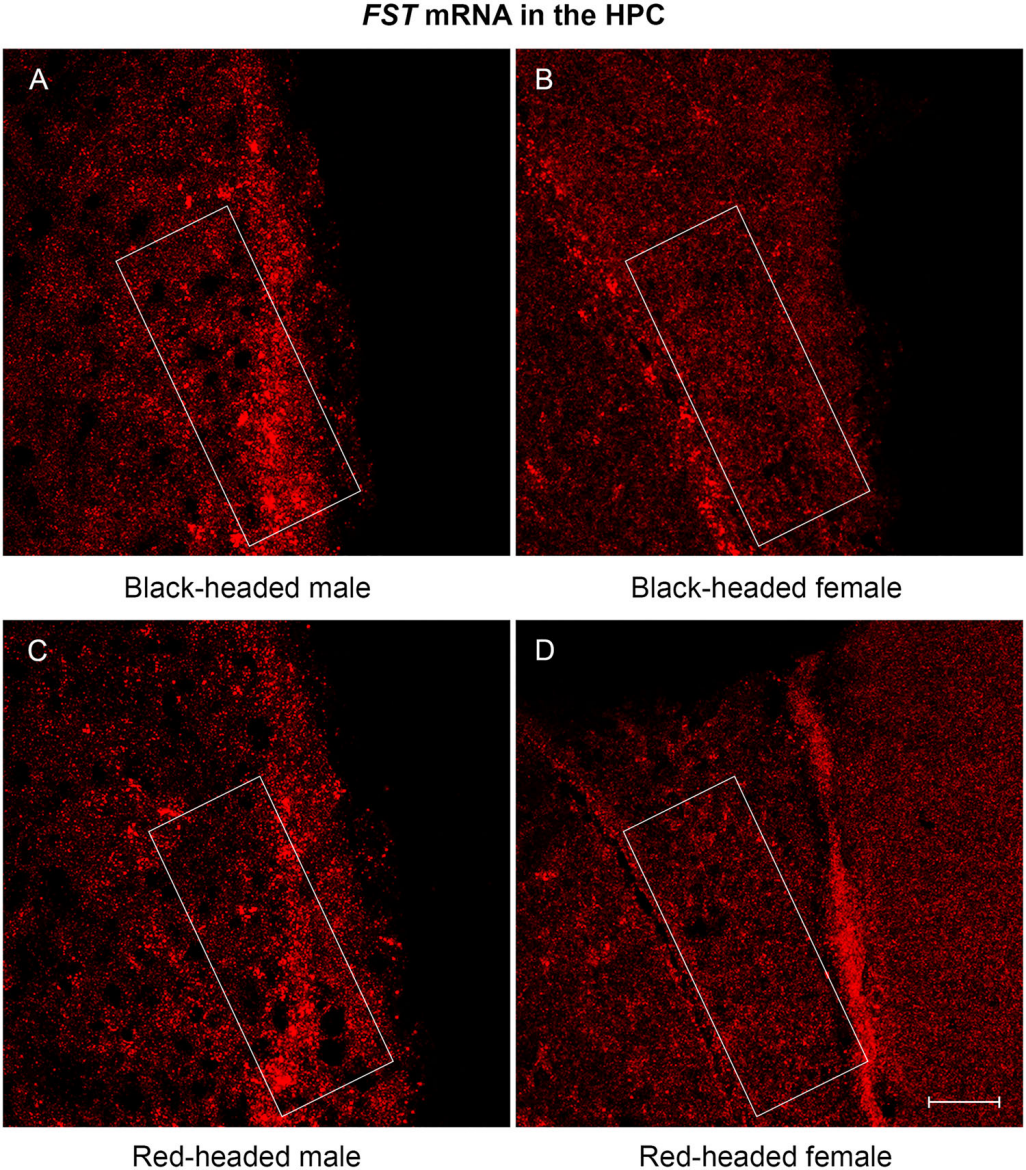
Representative photomicrographs of *FST* mRNA expression in the HPC of Gouldian finch brain. (A) Black‐headed male. (B) Black‐headed female. (C) Red‐headed male. (D) Red‐headed female. *FST* mRNAs were quantified in the white‐boxed areas. FST, follistatin; HPC, caudal hippocampal formation, hippocampus. Scale bar: 50 µm.

A moderate level of *FST* mRNA was expressed in the HPC of black‐ and red‐headed males (Figure 5A,C), whereas weak expression was detected in females (Figure 5B,D). It was evident that many dot‐like signals were concentrated in the triangular region of the HPC (Figure 5A,C). A two‐way ANOVA revealed a significant effect for sex, *F*(1, 12) = 41.68, *p* < 0.0001; but no significant effect for morph and interaction between sex and morph. In both males and females, *FST* mRNA in black‐headed birds did not differ from that of red‐headed birds (Figure 6J; both *p*‐values >0.05). However, in both black‐ and red‐headed birds, males displayed a higher level of *FST* mRNA than females (Figure 6J; *p* = 0.0004 for black‐headed, *p* = 0.001 for red‐headed).

FIGURE 6Quantitative analyses of *FST* mRNA expression in 10 brain regions involved in stress responses, aggression, and parental care of Gouldian finches. *FST* mRNA was assessed by the average punctate dot number per cell. (A) PVNL. (B) VMH. (C) BSTM. (D) BSTL. (E) HPR. (F) NHpC. (G) PAG. (H) VTA. (I) TnA. (J) HPC. ns *p* > 0.05 (not significant), *p < 0.05, **p < 0.01, ***p <  0.001, and ****p < 0.0001.

**Figure:**
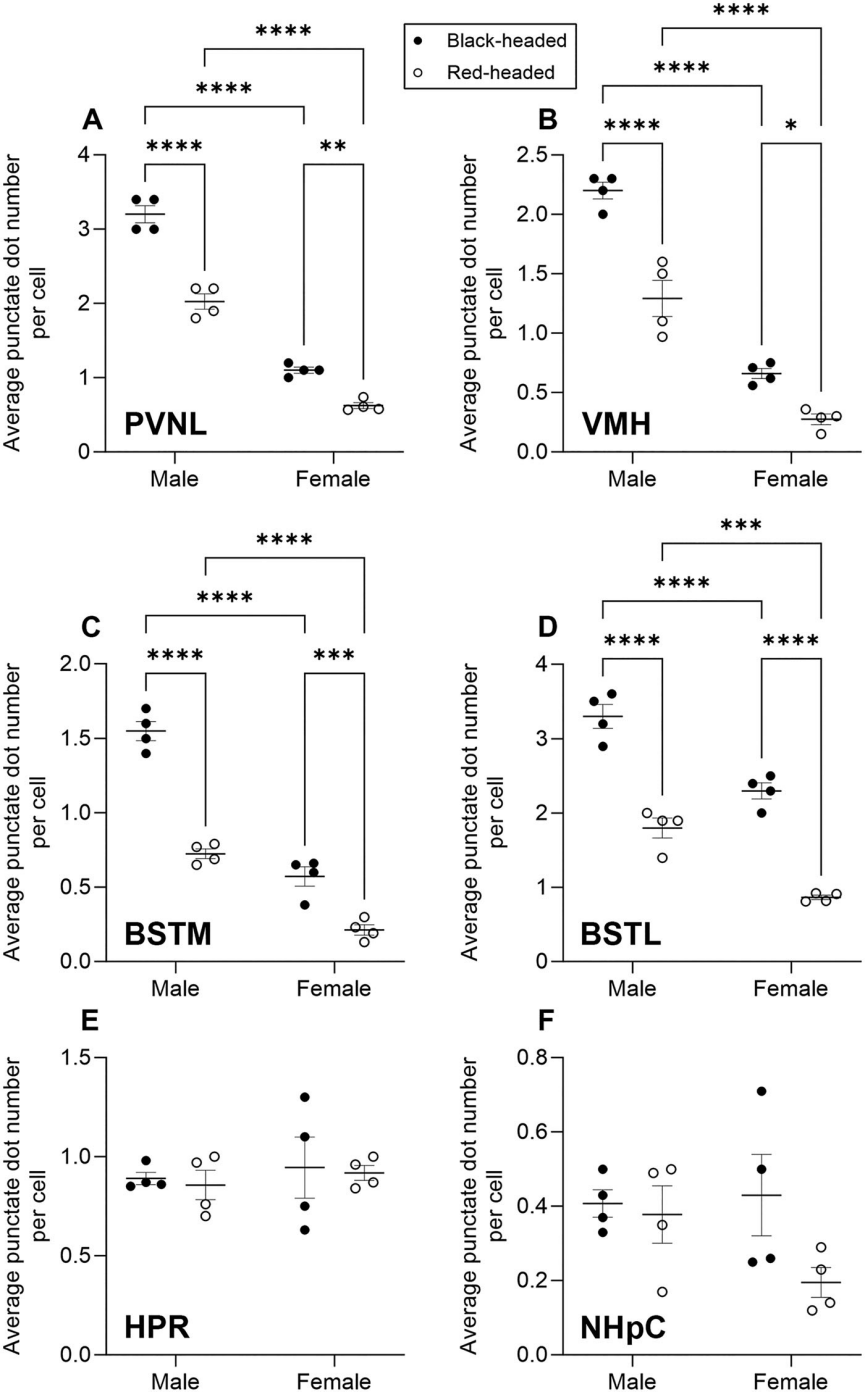

**Figure:**
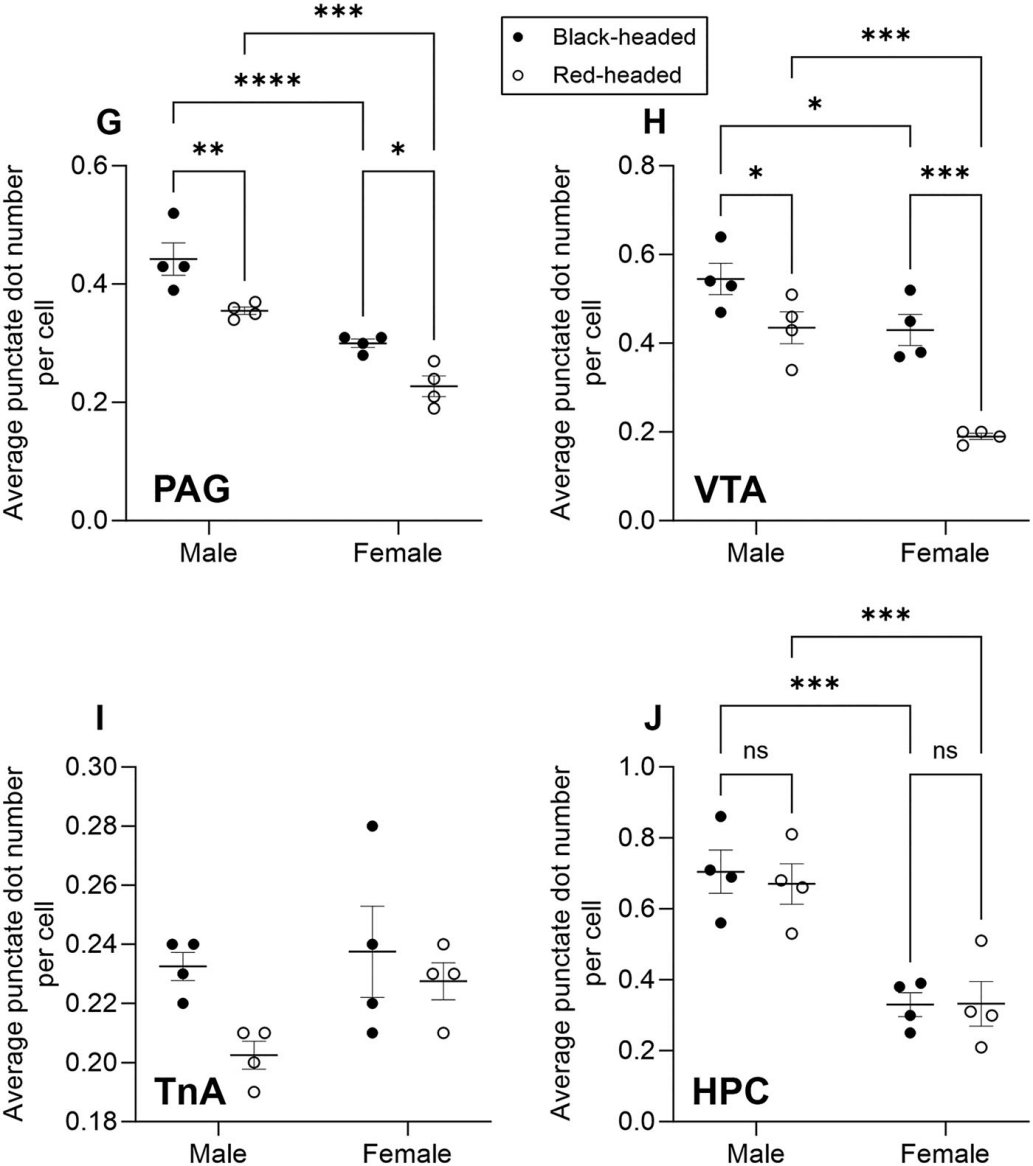

## 4 Discussion

Results of this study reveal a potentially important role for *FST* in social behaviors and support the hypothesis that differential expression of *FST* in the brains of red‐ and black‐headed morphs may function to regulate morph‐specific differences in multiple behavioral and physiological processes. We revealed for the first time in a songbird that *FST* mRNA was heterogeneously distributed across the brain, with the most abundant expression observed in the PVNL, VMH, and BSTL. Furthermore, *FST* mRNA expression differed significantly between morphs and sex in association with known behavioral and physiological differences. Black‐headed birds, irrespective of sex, exhibited greater levels of *FST* mRNA in many brain regions associated with aggression, stress responses, and parental care compared to red‐headed birds. Within the same morph type, males consistently expressed higher levels of *FST* mRNA than females.

### 4.1 *FST* and Morph‐Specific Differences in Aggression

In this study, we report morph‐specific differences in *FST* mRNAs in the BSTM, BSTL, VMH, and PAG (black‐headed > red‐headed), which are each considered components of a vertebrate social behavior network (Lischinsky and Lin 2020; Nelson and Trainor 2007). Studies in mammals demonstrate that each of these regions is involved in the production or modulation of aggressive behaviors (Lischinsky and Lin 2020; K. Hashikawa, Hashikawa, Tremblay, et al. 2017; Y. Hashikawa, Hashikawa, Falkner, et al. 2017; Lin et al. 2011; Falkner et al. 2016; Putkonen 1966; Lipp and Hunsperger 1978; Siegel and Pott 1988), and in songbirds, there is evidence that aggressive behaviors in response to intruders induce distinct patterns of immediate early gene activity in these regions (Goodson et al. 2005; Goodson and Evans 2004; Maney and Ball 2003). The localization of *FST* to these regions, along with morph‐specific expression patterns, suggests that *FST* perhaps modifies activity in social behavior networks to produce morph‐specific aggressive behaviors. As reviewed in the introduction, *FST* is perhaps best known for its role in the regulation of steroid hormones, such as testosterone (Bilezikjian et al. 1993; Besecke et al. 1997; DePaolo et al. 1993; Kamei et al. 2005), which may in part explain morph‐specific differences in aggressive behavior. However, the present findings suggest for the first time that *FST* may also influence behavior by acting directly in brain regions known to underlie aggressive and other social behaviors. Future studies are now needed to experimentally test the possibility that *FST* in the BSTM, BSTL, VMH, and PAG suppresses aggression in black‐headed morphs.

The nucleus taeniae of the amygdala (TnA) in birds is thought to be homologous to the medial amygdala in mammals and has been implicated in the regulation of aggressive behavior (Lischinsky and Lin 2020; Voigt et al. 2018). Despite its known role in aggression, we did not detect any morph‐ or sex‐related differences in *FST* mRNA expression within the TnA, suggesting that increased aggression in the red morph may be mediated via a different neural mechanism.

### 4.2 *FST* and Morph‐Specific Differences in Stress Responses

Similar to what has been reported in mammals (Macconell et al. 1996; Ogawa et al. 2020), *FST* in Gouldian finches was present in the PVN, VMH, and BST. For each of these regions, *FST* mRNA expression was higher in black‐headed compared to red‐headed birds. Across vertebrates, the PVN is the brain control center for regulating stress responses (Nagarajan et al. 2014; Kuenzel et al. 2020; Herman et al. 2016). It regulates corticosterone release by secreting corticotropin‐releasing hormone (CRH) into the hypophyseal portal system, resulting in the downstream release of glucocorticoids (i.e., corticosterone in birds) from the adrenal glands. As previously mentioned, FST carries out most of its biological functions in the CNS by inhibiting the activity of the protein activin (Patel 1998; Phillips and de Kretser 1998). Past studies in mammals demonstrate that activin in the PVN facilitates CRH secretion (Plotsky 1991). The finding that *FST* mRNA was significantly higher in the PVNL of black‐headed compared to red‐headed males in the present study suggests that FST may act to inhibit activin‐induced release of CRH in black‐headed birds. This may explain previously reported morph differences in corticosterone release in response to social challenges (red‐headed > black‐headed) (Pryke et al. 2007). In addition, projections to the PVN from both the BST and VMH can also modulate stress responses and glucocorticoid release (Daniel and Rainnie 2016). It is likely that FST in the BST and VMH regulates stress responses via their projections to the PVN by a similar way, inhibiting activin‐induced release of CRH, although to our knowledge the role of activin in these regions has not been studied. Future studies are now needed to experimentally test the possibility that *FST* in the PVN, BSTL, BSTM, and VMH suppresses stress responses in black‐headed morphs.

### 4.3 *FST* and Morph‐Specific Differences in Parental Care

Red and black females provided contrasting parental care to their offspring when receiving a low‐quality diet. Specifically, red females significantly reduced the frequency with which they fed their fostered offspring, whereas black females significantly increased their provisioning rates (Pryke et al. 2012). Similarly, red males significantly reduced their visitation rates, while black males significantly increased their provisioning rates (Pryke et al. 2012). These findings suggest that FST levels may be positively correlated with parental efforts.

The PVN and BST, along with the PAG, VTA, and TnA, are also part of neural networks involved in parental care, in which distinct patterns of activation are proposed to underlie distinct components of parental behaviors (Numan and Insel 2003). These brain regions are densely interconnected and interact with each other by processing aspects of perception and sensory processing, neural coordination, motivation, movement, and advanced cognitive regulation (Kohl and Dulac 2018; Yu et al. 2020; Dulac et al. 2014). In this study, we report differences in *FST* mRNA in the BSTM, BSTL, VMH, PAG, and VTA for morphs (black‐headed > red‐headed), which map onto previous studies showing that black‐headed birds exhibit more parental behavior in competitive environments than red‐headed birds (Pryke and Griffith 2009; Pryke et al. 2012). Future studies are now needed to determine the degree to which *FST* and activin in these regions contribute to morph‐specific differences in parental behavior.

### 4.4 Sex Differences in *FST*

Various animals, including mammals, fish, and birds, display sex differences in multiple physiological, psychological, and behavioral processes such as aggression (Archer 2004), parental care (La Mesa et al. 2021; Lonstein and De Vries 2000; Liker et al. 2015), and stress responses (Sterrenburg et al. 2012). In this study, in addition to morph‐specific differences, we identified sex‐specific expression patterns of *FST* mRNAs in brain regions associated with aggression, parental care, and stress responses, including the VMH, PVNL, BSTM, BSTL, PAG, VTA, and HPC. Notably, males consistently exhibited higher levels of *FST* mRNAs compared to females within the same morph.

The *FST* gene is located on the Z chromosome. In birds, males are ZZ and females are ZW, and unlike mammals, birds generally lack global sex chromosome inactivation (McQueen et al. 2001). Consequently, one might expect to see higher expression of Z‐linked genes like *FST* in males than in females. Consistent with this expectation, we observed significantly higher *FST* mRNA levels in males as compared to females within the same morph across several brain regions, including the VMH, PVNL, BSTM, BSTL, PAG, VTA, and HPC. However, previous studies have shown that most Z‐linked genes, including *FST*, are subject to dosage compensation in birds, which would typically equalize gene expression between sexes (McQueen et al. 2001). The male‐biased expression of *FST* in the aforementioned brain regions suggests an absence of effective dosage compensation. In contrast, in two other brain regions—the HPR and TnA—we observed no significant difference in *FST* expression between males and females, indicating that dosage compensation may occur in these areas. The mechanisms underlying regional variation in dosage compensation of *FST* expression in the Gouldian finch brain remain unclear.

While previous research has focused almost exclusively on the differences between head color morphs in the Gouldian finch (Pryke and Griffith 2009; Pryke et al. 2012; Williams et al. 2012), relatively little is known about the sex differences in endocrine, physiological, and behavioral traits within a single morph. Currently, the functional significance of these sex‐specific differences in *FST* mRNA expression remains unclear. Future studies should be conducted to explore whether and how these differences influence sex‐specific psychological, physiological, and behavioral processes in Gouldian finches.

### 4.5 Other Considerations and Limitations

FST has been shown to play a protective role in response to various forms of physiological and physical stress, including oxidative stress, glucose deprivation, ionizing radiation, and shear stress (Zhang et al. 2018). In addition to its cytoprotective functions, FST also influences anxiety‐related behavior; for example, mice overexpressing FST display increased anxiety‐like behaviors (Ageta et al. 2008). FST protein is widely distributed throughout the rodent brain (Ogawa et al. 2020; MacConell et al. 1998). *FST* gene‐specific knockdown in the rodent hippocampus decreases neurogenesis and impaired learning and long‐term potentiation (Chen et al. 2021).

Social behaviors such as aggression and parental care in songbirds are differentially expressed across the annual cycle. Aggression typically peaks during the period of territory and mate acquisition and declines during parental phases (Wingfield et al. 1990; Watts 2020). The seasonal plasticity of these behaviors has been associated with changes in circulating hormone levels and sex steroid receptor expression (Watts 2020). One would expect a relatively stable circulating hormone level in this study as birds were housed in a nonreproductive, socially neutral context. Although behavioral data were not recorded, birds were monitored daily for aggression, and we did not observe birds displaying high levels of aggressive behaviors. Thus, the gene expression profiles demonstrated in this study are baseline levels rather than reflecting the highest or lowest behavior expression.

The mechanisms underlying the color morph‐specific expression of *FST* in the brain remain unexplored. It has been proposed that a candidate regulatory region within the *Red* locus, located upstream of *FST*, may drive the spatiotemporal variation in its expression, as no differences have been found in the *FST* gene or protein‐coding sequences between black‐ and red‐headed birds (Kim et al. 2019; Toomey et al. 2018). Differences in transcription factor binding sites within the *Red* locus—present in one morph but not the other—could potentially account for the morph‐specific expression of *FST*. Future studies are needed to identify the structural differences in this regulatory region between morphs.

One limitation of the present study is that it remains unclear if the head color morph‐ and sex‐specific differences in *FST* gene expression within brain regions examined are unique to this gene. Preliminary data from an ongoing project in the lab investigating the expression of the activin receptor gene (ACVR2A) strongly support the idea that these differences are gene specific. As identified, ACVR2A mRNA levels did not differ significantly between morphs or sexes in the BSTL, a region affecting song variability, performance, and stress responses in songbirds (Nagarajan et al. 2014; Smulders 2021) (Figure S1).

This study demonstrated that *FST* mRNA is heterogeneously distributed throughout the Gouldian finch brain, with significant differences observed between morphs and sexes. A key question arising from these findings is whether the observed differences are due to increased expression per cell or an increased number of *FST*‐expressing cells. Our results support the former mechanism—higher expression per cell—since the number of *FST‐*expressing cells in most target regions did not significantly differ between morphs or sexes. However, we cannot entirely rule out the possibility of increased cell numbers contributing to the differences in specific regions, such as the VMH, NHpC, and HPC (see Table 1).

## 5 Conclusion

In summary, our findings suggest that the head color morph‐ and sex‐specific differences in *FST* gene expression in the Gouldian finch brain may underlie the observed morph‐ and sex‐specific variations in aggression, stress responses, and parental care. Our results support the hypothesis that differential expression of *FST* in the brain in red‐ and black‐headed morphs might be a genetic mechanism through which *FST* influences multiple behavioral and physiological processes. Additionally, our results enhance our understanding of the genetic and neuroendocrine mechanisms underlying behavioral polymorphisms and provide valuable insights for exploring social behavior, neuroplasticity, and stress regulation in other systems. Future studies are now needed to manipulate *FST* and activin in each brain region to determine their contributions to morph‐ and sex‐specific social behaviors and endocrine responses.

## Author Contributions

C.Z. and F.M. conceived and designed the study, analyzed data, and drafted and revised the manuscript. C.Z. performed experiments.

## Conflicts of Interest

The authors declare no conflicts of interest.

## Peer Review

The peer review history for this article is available at https://publons.com/publon/10.1002/cne.70098.

## Supporting information




**Figure S1** Representative photomicrographs of the activin receptor gene (ACVR2A) mRNA expression in the BSTL of Gouldian finch brain. A, black‐headed male; B, black‐headed female; C, red‐headed male; D, red‐headed female. Note that ACVR2A mRNAs did not differ significantly between morphs or sexes in the BSTL (the white boxed areas). Abbreviations: BSTL, lateral bed nucleus of the stria terminalis. Scale bar: 50 µm.

## Data Availability

The data supporting the findings of this study are openly available in DRYAD at https://doi.org/10.5061/dryad.7pvmcvf5x.
