# Supplementary figures and images for: Head Color Morph‐ and Sex‐Specific Differences in Follistatin Gene Expression in the Gouldian Finch Brain
Source: J Comp Neurol. 2025 Oct 17;533(10):e70098. doi: 10.1002/cne.70098 (PMC12534724; doi:10.1002/cne.70098)

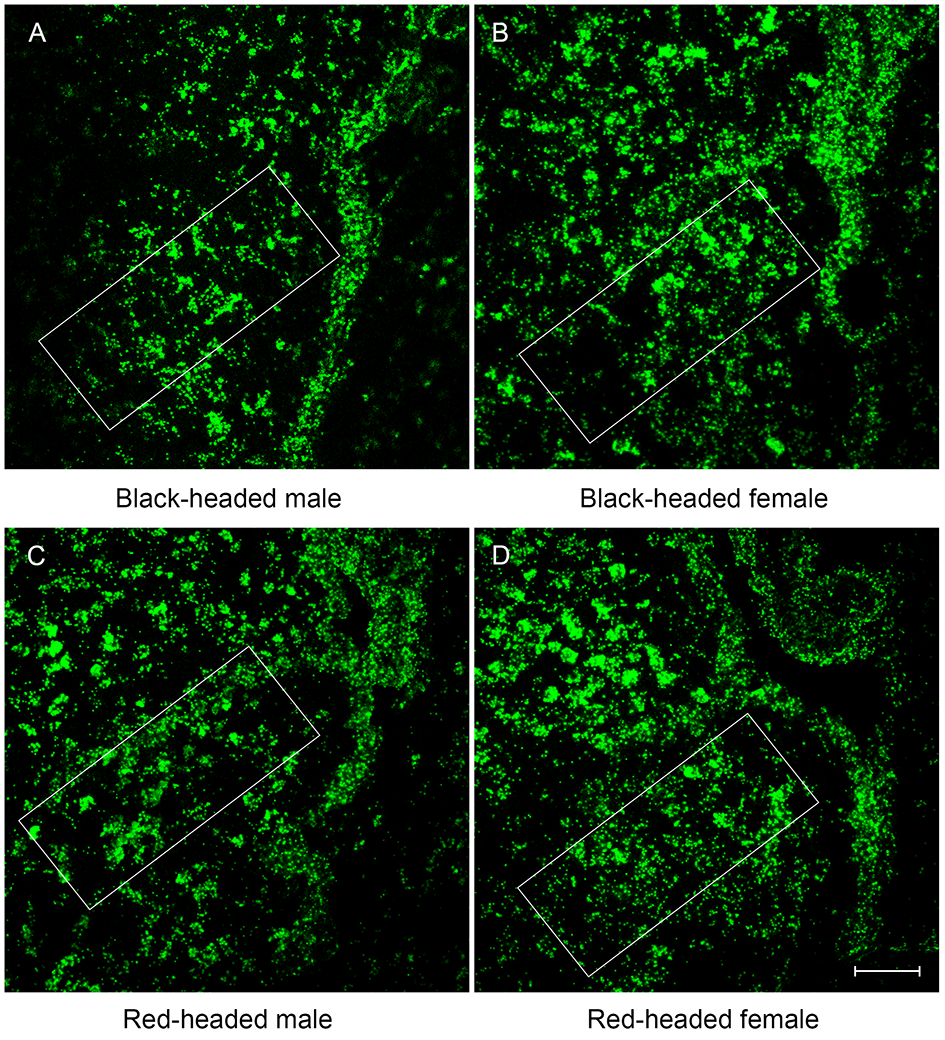

Supplement: Supplementary file 1 — Figure S1 Representative photomicrographs of the activin receptor gene (ACVR2A) mRNA expression in the BSTL of Gouldian finch brain. A, black‐headed male; B, black‐headed female; C, red‐headed male; D, red‐headed female. Note that ACVR2A mRNAs did not differ significantly between morphs or sexes in the BSTL (the white boxed areas). Abbreviations: BSTL, lateral bed nucleus of the stria terminalis. Scale bar: 50 µm. [file CNE-533-e70098-s001.tif]
